# Special Issue Editorial: “Antibacterial Agents from Natural Source, 2nd Edition”

**DOI:** 10.3390/molecules31173077

**Published:** 2026-09-01

**Authors:** Carlos Henrique Gomes Martins

**Affiliations:** Laboratory of Antimicrobial Testing, Department of Microbiology, Institute of Biomedical Sciences, Federal University of Uberlândia, Uberlândia 38405320, Minas Gerais, Brazil; carlos.martins2@ufu.br; Tel.: +55-34-3239-1363

Antimicrobial resistance remains one of the most pressing challenges for global health, affecting human, animal, and environmental health. The continuous emergence of multidrug-resistant bacteria, together with the limited discovery of new conventional antibiotics, has intensified the search for alternative, effective, and sustainable antibacterial strategies. In this context, natural products and nature-inspired compounds remain valuable and promising sources of new antibacterial agents.

The Special Issue “Antibacterial Agents from Natural Source, 2nd Edition” was conceived to bring together studies focused on the discovery, characterization, and biological evaluation of antibacterial compounds obtained from natural sources. This second edition includes five original research articles and two review articles, reflecting both experimental advances and critical syntheses of current knowledge in the field. Together, these contributions reinforce the relevance of plants, fungi, peptides, proteins, secondary metabolites, and nanostructured systems as important platforms for the development of novel antibacterial, antibiofilm, and antivirulence strategies.

The original research articles published in this collection highlight the diversity and richness of natural sources in antibacterial research. Okińczyc et al. investigated the phytochemical profiles and antimicrobial activity of selected *Populus* spp. bud extracts, demonstrating relevant activity especially against Gram-positive bacteria and *Helicobacter pylori*. The observed antibacterial effects were associated with the presence of flavonoids, hydroxycinnamic acid esters, and other phenolic compounds, highlighting *Populus* buds as a promising source of bioactive molecules.

Gao et al. explored the chemical constituents of the arils of *Torreya grandis*, reporting abietane-type diterpenoids, including previously undescribed compounds. Some of these metabolites showed inhibitory activity against methicillin-resistant *Staphylococcus aureus*, reinforcing the importance of natural product chemistry in the identification of structurally diverse antibacterial prototypes.

Bolaños-Nuñez et al. described *Fulvifomes mexicanus* sp. nov. and investigated its antipathogenic potential. The methanolic extract of this fungus inhibited bacterial motility and quorum sensing-associated phenotypes, suggesting that natural products may act not only by inhibiting bacterial growth but also by attenuating virulence mechanisms. This antivirulence approach represents an important strategy in the search for new antibacterial agents.

Haralambie et al. evaluated ethanolic extracts of *Origanum majorana*, *Salvia officinalis*, and *Ribes nigrum* against digestive bacterial pathogens, and their findings demonstrated bactericidal activity and relevant polyphenolic content, supporting the potential application of these plant extracts as natural antibacterial agents or as adjuvants to conventional antimicrobial therapy.

Cabral et al. investigated the synergistic antibacterial effect of eugenol and biogenic silver nanoparticles against multidrug-resistant *Staphylococcus pseudintermedius* isolated from canine keratoconjunctivitis sicca. The combination showed activity against planktonic and biofilm-associated cells, promoted structural changes in bacterial cells, and presented reduced toxicity to mammalian cells under the tested conditions. This contribution highlights the potential of combining natural compounds and nanotechnology to enhance antibacterial efficacy.

In addition to these original investigations, this Special Issue includes two review articles that broaden the conceptual and translational discussion on natural antibacterial strategies. Yang et al. reviewed the role and mechanisms of antimicrobial peptides in overcoming multidrug-resistant bacteria. Antimicrobial peptides are particularly attractive because of their broad-spectrum activity, membrane-targeting mechanisms, immunomodulatory effects, and potential synergism with conventional antibiotics, and this review emphasizes their relevance as promising candidates for future antibacterial therapies.

Napoleão et al. reviewed the potential of lectins as natural antibiofilm agents in the fight against antibiotic resistance. Since biofilms are highly tolerant to antimicrobial agents and immune responses, the identification of natural molecules capable of inhibiting biofilm formation or disrupting established biofilms is of great relevance. This review reinforces the importance of lectins as promising antibacterial and antibiofilm tools, while also identifying important gaps for future investigation.

Together, the contributions included in “Antibacterial Agents from Natural Source, 2nd Edition” demonstrate that natural sources continue to offer a broad and chemically diverse repertoire of molecules with antibacterial potential. The five original articles provide experimental evidence on bioactive extracts, isolated compounds, fungal metabolites, natural compound–nanoparticle combinations, and antibacterial activity against clinically and veterinary relevant pathogens. The two review articles complement this experimental framework by critically discussing antimicrobial peptides and lectins as emerging natural strategies against multidrug-resistant bacteria and biofilm-associated infections.

These studies also show that the development of new antibacterial agents must consider not only direct antimicrobial activity, but also antibiofilm effects, antivirulence mechanisms, synergistic combinations, toxicity, and translational applicability. The findings presented in this collection reinforce the need for integrated approaches combining natural product chemistry, microbiology, pharmacology, biotechnology, nanotechnology, and molecular studies. Future research should prioritize the standardization of extracts, identification of active compounds, elucidation of mechanisms of action, evaluation of toxicity, in vivo validation, and the development of formulations suitable for clinical, veterinary, agricultural, or industrial applications.

As Guest Editor, I would like to sincerely thank all authors for their valuable contributions to this Special Issue and all reviewers for their careful and constructive evaluations. I also acknowledge the editorial team of *Molecules* for their continuous support throughout the editorial process.

